# Supplementary material for: Exploring providers’ perceived barriers to utilization of antenatal and delivery services in urban and rural communities of Ebonyi state, Nigeria: A qualitative study
Source: PLoS One. 2021 May 20;16(5):e0252024. doi: 10.1371/journal.pone.0252024 (PMC8136846; doi:10.1371/journal.pone.0252024)
Supplement: S1 File — (DOCX) [file pone.0252024.s001.docx]

**KEY INFORMANT INTERVIEW Guide**

Greetings

Introductions

Opening formalities

Obtain consent and seek for permission to record the interview

**Instructions for Interviewer**

After beginning the audio recording, start interview recoding by stating the date, time and name of interviewer.

Ask for basic information concerning the participant. (Participant profile).

Interview guide

1. I am going to ask you some questions on utilization of health facilities for antenatal and delivery services. How do you rate the utilization (attendance) of Antenatal care and delivery services in your health center? There should be an answer, Good, Fair, Poor, etc
2. What are the reasons for the current utilization rates? (based on the answer above)
3. Are you satisfied with the utilization of ANC and delivery services in your health facility? Yes/No. Why?
4. What is your view about the state of equipment/facilities/medicines for providing antenatal care and delivery services in your health facility?
5. How do you rate the staff strength of your facility? Are you overworked?
6. Does your health facility offer 24-hour service? YES/NO. Why? Does it affect delivery of services in your health facility? Give reasons.
7. What factors do you think make women not to attend ANC in formal health facilities?
8. What factors do you think make the women not to deliver in health facilities even after attending antenatal care there?
9. What are the reasons why women deliver at home?
10. What are the reasons women deliver their babies with traditional birth attendants?
11. What are the difficulties/constraints in assessing ANC in health facilities?
12. What are the difficulties women encounter in delivering their babies in health facilities?
13. What do you think can be done to overcome these difficulties. (for both questions 11 and 12)
14. What are the roles of health workers in ensuring that women attend ANC and deliver in health facilities?
15. Do husbands have a role to play in determining where women attend ANC and where they deliver their babies? (Yes or No) then explain.
16. What are the roles of Government at all levels in ensuring that women attend ANC and deliver in health facilities?

.
