# Supplementary material for: Exploring providers’ perceived barriers to utilization of antenatal and delivery services in urban and rural communities of Ebonyi state, Nigeria: A qualitative study
Source: PLoS One. 2021 May 20;16(5):e0252024. doi: 10.1371/journal.pone.0252024 (PMC8136846; doi:10.1371/journal.pone.0252024)
Supplement: S2 File — (DOCX) [file pone.0252024.s002.docx]

**Introduction**

1. **How do you rate the utilization (attendance) of Antenatal care and delivery services in your health center? There should be an answer, Good, Fair, Poor, etc**

**Ans. –** I will grade it good because Health Centres and Private Clinics are everywhere, but they still are to this centre our turning up is okay.

1. **What are the reasons for the current utilization rates?**

**Ans. –** One major reason for this good turn up in women using this centre for ANC and delivery is because of this program MCHCP – material and child healthcare programe that came. The government called us for training and give us materials. They even though us what those in secondary level (i.e. General Hospitals) are doing, so we can do it in health centres and be able to save mothers and their children. There is a good change in the turn up as we are now able to do what specialist do.

1. **Are you satisfied with the utilization rates for ANC and delivery? Yes/No. Why?**

**Ans. –** I am not very satisfied, I still want more women to come for ANC and deliver in this facility.

1. **What is your view about the state of equipment/facilities/medicines for providing antenatal care and delivery services in your health center?**

**Ans. –** We have enough just that our delivery cutch spoilt. We are now using table to improvise. Also we need a staff Quarter where staffs can stay (live) for more effective work.

1. **How do you rate the staff strength of your facility? Are you overworked (understaff)?**

**Ans. –** We are short staffed. So we need more qualified staff.

1. **Does your health facility offer 24 hour service? YES/NO. Why? Does it affect delivery in the health center? Give reasons.**

**Ans. –** Yes – so every time, somebody is here.

1. **What factors do you think make women not to attend ANC in formal health facilities?**

**Ans. –** People that are already with private practitioners.

**Are the private practitioners trained?** – Yes, many of the health workers who have no jobs are practicing in their private homes. Since the MCHCP started, the Health Committee of the village and chiefs are taught on the importance of using trained health workers, people are now understanding and ANC has increased to about 75%.

**Interview –** Do people still you FBAs:

**Ans.** – There is only one renown TBA in this place and she is now part of the Health Committee, but people are no longer patronizing her. Since I came, nobody has delivered there. Even one pregnant women who lives besides her, she brought the women to Health Centre by herself.

1. **What do you think make the women not to deliver in health facilities even after attending antenatal care there?**

**Ans. –** Since I came, people who registered with us for ANC all delivered with us except one primp who came and was asked to go back because she was not in actual labour. She then travelled to another community far away and stayed some days and eventually delivered there.

1. **What are the reasons why women deliver at home?**

**Ans.1 -** When they don’t have money to pay for the facility services.

**2 –** If labour started and they ignore it until the head of the baby is almost at the perineum before they start preparing for Hospital, labour is not a sudden event. So if the person does not watch out well.

**V.I.P –** The TBA in the community was made part of the Health Committee and she became involved in referring people to the health centre.

1. **What are the reasons women deliver their babies with traditional birth attendants?**

**Ans. –** Women register in more than one place in case of referral. We encourage women who register with us to also register in General Hospital in case of complication, so we can refer them where they will be attended to with ease.

1. **What are the difficulties women encounter in delivering their babies in health facilities?**

**Ans. 11-13 –** Money – Is the major constraint, because despite the fact that we only charge very little amount, some people still cannot afford it.

**-** Distance is not a major constraint because the centre is located at the centre of the town and if you have money, you can go anywhere.

1. **What can be done to overcome these difficulties. (both ANC and DS)**

**Ans. –** If the government can provide everything needed for ANC and delivery care – solutions, drugs, materials etc and thus no need to give mothers list of items to buy.

**-** Also if the government can provide jobs for the citizens, so they can have something doing and thus able to afford them healthcare bills. Empowering women especially in small business will be a great way out.

1. **What are the roles of health workers in ensuring that women attend ANC and deliver in the health centre?**

**Ans. –** Home visit

**-** Creating good rapport with the women that come to the facility for care.

- Being on ground 24hours so that when the women come they will have confidence and come.

1. **Do husbands have a role to play in determining where women attend ANC and where they deliver their babies? (Yes or No) then explain.**

**Ans. –** Yes, for now, they said that husbands should accompany their pregnant wives to the 1^st^ ANC visit so that they too can listen to the health talks e.g. on issues affecting the women and her baby, for instance, that a pregnant woman can eat eggs, snails etc and nothing negative will happen to the baby. Also to encourage the family (Husband) to start saving for delivery from the 1^st^ month of pregnancy and also to start giving the woman small amount per time to buy things ahead of time.

**Interview –** How do you get the men to come?

**Ans.** – We used to tell any husband to come to drop their wife (or anywhere we see them) about the recent WHO (World Health Organization) policy that husband should be part of ANC.

1. **What are the roles of Government at all levels in ensuring that women attend ANC and deliver in health centre?**

**Ans. –** Creating jobs/empowering mothers

**-** We will not say that government should make ANC and facility delivery a law because it will not work.

**Conclusion –**

**Introduction**

1. **How do you rate the utilization (attendance) of Antenatal care and delivery services in your health center? There should be an answer, Good, Fair, Poor, etc**

**Ans. –** It is not all that poor. They use to patronize it but not to its fullness, there are still people that attend outside like TMS.

1. **What are the reasons for the current utilization rates?**

**Ans. –** The reasons why people do not really patronize it are because of the access fee confirmation, the distance of the health centre to them, i.e. if the health centre is far. The commonest reason is that they don’t see new people if they come. Again is the rate of attendance, they don’t like to be delayed. They always want to be attended to immediately they arrive. They don’t want to wait for anything even health talks as they complain that they are going to farms, market etc. To them you are wasting their time.

1. **Are you satisfied with the utilization rates for ANC and delivery? Yes/No. Why?**

**Ans. –** NO, because when the targeted population are not gotten, they weaken you and they suppose to be many people coming for antenatal care but they are not coming.

1. **What is your view about the state of equipment/facilities/medicines for providing antenatal care and delivery services in your health center?**

**Ans. –** Everything is well equipped by donor agencies e.g. ICC, CCM and population even at that the population of attendants is still not as expected. Even the delivery equipments are in place. There even more equipments than is need in the place, many are too big for a small hospital like this and are just lying waste.

1. **How do you rate the staff strength of your facility? Are you overworked (understaff)?**

**Ans. –** Not understaffed. Many staff were posted.

1. **Does your health facility offer 24 hour service? YES/NO. Why? Does it affect delivery in the health center? Give reasons.**

**Ans. –** Yes, we run 24hours services, so people can come to deliver at anytime.

1. **What factors do you think make women not to attend ANC in formal health facilities?**

**Ans. –** They say the staff are not always on duty.

**(b)** The distance from their homes.

**(c)** And attending to them quickly whenever they come

**(d)** Financial aspect – They complain that the former Officer Incharge bills them high but since I came, price has reduced.

1. **What do you think make the women not to deliver in health facilities even after attending antenatal care there?**

**Ans. –** They are many that prefer delivering in other places after attending ANC here. They prefer TBA, they give excuses that the staff are not always available. They prefer delivering in their homes.

1. **What are the reasons why women deliver at home?**

**Ans. –** Some deliver at home because of financial problems. They said that TBAs allows them to pay installmentally. They complain also that staff are not present. The same with ANC but worse in delivery.

1. **What are the reasons women deliver their babies with traditional birth attendants?**

**Ans. –** The same reasons that made them deliver at home make them deliver at TBAs. Because they need to be educated on the need to attend ANC and delivery in Health Centres. Even if you make it free, some will still deliver at home.

1. **What are the reasons why women attend antenatal care in more than one health facility?**

**Ans. –** Women attend ANC in more than one place like TBAs, chemist stores, etc. But when their cases become difficult, they refer them to Health Centres.

1. **What are the difficulties/constraints in assessing ANC in health facilities?**

**Ans. –** The difficulties in getting ANC here are finance, distance and false perception about health workers.

1. **What are the difficulties women encounter in delivering their babies in health facilities?**

**Ans. –** To overcome these difficulties both in ANC and delivery the staff will have to sensitize the women in their villages, carrying out enlightenment programmes. Village chiefs have always promosed to have meeting with us and women but yet to do. Let hope it will be possible.

1. **What can be done to overcome these difficulties. (both ANC and DS)**

**Ans. –** Health workers can carryout sensitization programmes and encourage them to come.

1. **What are the roles of health workers in ensuring that women attend ANC and deliver in the health centre?**

**Ans. –** Home visit and even enlighten them in churches, at women gatherings and some things of sort.

1. **Do husbands have a role to play in determining where women attend ANC and where they deliver their babies? (Yes or No) then explain.**

**Ans. –** Husbands have roles to play in their ANC and DS. They do this through advising their wives. So if men can be reached to advise their wives to attend ANC.

1. **What are the roles of Government at all levels in ensuring that women attend ANC and deliver in health centre?**

**Ans. –** The government contributes to ANC and DS by giving free medical care to help solve the financial problems.

**Conclusion**

**Introduction**

1. **How do you rate the utilization (attendance) of Antenatal care and delivery services in your health center? There should be an answer, Good, Fair, Poor, etc**

**Ans. –** The lady who have headed the clinic for 3years said that the attendance is not that poor and that they held ANC every Monday (i.e. once in a week) and many women attend. However, she said that although they are trying, the delivery is relatively poor since in a month they have about 4, 5, or 6 deliveries in the clinic.

1. **What are the reasons for the current utilization rates?**

**Ans. –** She pointed out monetary aspect as one of the reasons why the utilization rate is not too good. Also family planning affects the utilization of ANC, which also reduced the number of women getting pregnant and number of women giving birth.

1. **Are you satisfied with the utilization rates for ANC and delivery? Yes/No. Why?**

**Ans. –** Yes because we were told to encourage and counsel them on family planning and many of the women are embarking family planning. The more woman on family planning, the less they utilize ANC and delivery services.

1. **What is your view about the state of equipment/facilities/medicines for providing antenatal care and delivery services in your health center?**

**Ans. –** The place is not properly equipped, in fact everything am using here is my personal equipment. However, equipments are not affecting the services.

1. **How do you rate the staff strength of your facility? Are you overworked (understaff)?**

**Ans. –** She said that she is the only staff in the centre and does all the work. In other words, she is overworked.

1. **Does your health facility offer 24 hour service? YES/NO. Why? Does it affect delivery in the health center? Give reasons.**

**Ans. –** It does not affect 24hours services much since my residence is here.

1. **What factors do you think make women not to attend ANC in formal health facilities?**

**Ans. –** Few people that are pregnant are coming but are still few because of family planning. Some people deliver in health centre while some still deliver at home. For instance, two women that were coming for ANC, while one of them delivered at the centre, one tried delivering at home but the case became critical and had to be rushed to the hospital. People are coming and they deliver here unless the person has problem, then she will be referred to the General Hospital.

1. **What do you think make the women not to deliver in health facilities even after attending antenatal care there?**

**Ans. –** I don’t know why they should attempt delivering at home even after attending ANC.

1. **What are the reasons why women deliver at home?**

**Ans. –** I don’t know.

1. **What are the reasons women deliver their babies with traditional birth attendants?**

**Ans. –** It is not a trend and that some people still deliver at TBAs, giving instance of a woman who attempted delivering at TBA but was later rushed to the General Hospital due to complication.

1. **What are the reasons why women attend antenatal care in more than one health facility?**

**Ans. –** The reason is because there are limited equipment in the health centre, so the women are encouraged to register also in a well equipped hospital with experts in case of complication or emergency.

1. **What are the difficulties/constraints in assessing ANC in health facilities?**

**Ans. –** I don’t see difficulties because we are not charging much and although is far, it is not too far from people. Anybody that wants to come the roads are accessible and okadas are there.

1. **What are the difficulties women encounter in delivering their babies in health facilities?**
2. **What can be done to overcome these difficulties. (both ANC and DS)**

**Ans. -** Women can be assisted with their transport fees. Most of them use okada to come for ANS and to pay their bills in case of delivery services. Also empowering women with minor jobs.

1. **What are the roles of health workers in ensuring that women attend ANC and deliver in the health centre?**

**Ans. –**

1. “Immobilization” **–** It will help them know some certain things to do and understand the necessity of ANC.
2. Public enlightenment and home visit to help know the need why they should come for ANC. The visit which also includes during village meeting will help them know those that are pregnant, and then encourage them. And also inform them that someone is always there in the health centre to take care of them.
3. **Do husbands have a role to play in determining where women attend ANC and where they deliver their babies? (Yes or No) then explain.**

**Ans. –** Yes! We are encouraging them to be helping their wives and even to accompany them to the clinics. Because it gives women joy when their husbands accompany them to the hospital or health centre.

It also affords men opportunity to learn from the health talks on who to care for their pregnant wives, save for the baby and how to handle issues on health.

1. **What are the roles of Government at all levels in ensuring that women attend ANC and deliver in health centre?**

**Ans. –** They should provide free medication as well as delivery packs to less the cost of ANC and delivery. Also equipping the centres with all that is need to run it to ensure women are not charged.

**Conclusion**

**Introduction**

1. **How do you rate the utilization (attendance) of Antenatal care and delivery services in your health center? There should be an answer, Good, Fair, Poor, etc**

**Ans. –** Poor or very poor, from my understanding these factors play out.

1. Ignorance bordering on the fact that some of the women still believe on the modern ways forefathers did it. They still don’t know the difference between those who deliver in the hospital.
2. Poverty – very serious. Villagers are poor, uneducated and unemployed. Even if someone wants to come to Hospital for ANC or any other reason, transport fee is still a big problem.
3. Proliferation of TBAs, patent medicine dealers (chemist) – people who wish to come to hospital are most of the time intercepted and diverted to chemist shops.
4. Unavailability of “good” health centres of hospitals to the people in where they live. This is because many of these people live in the winter lands far away and find it difficult to come out. So if there is a way to take medicare to them, it will be good.
5. Attitude of health workers. Even in the few available hospitals, workers are rarely seen on duty or when they come, their attitudes to the patient discourages them. This makes them loose confidence on the health facilities and resort to TBAs and chemist shops.
6. On government side, unavailability of some basic drugs in the hospital stores. Imagine if a patient comes to the hospital and you prescribe drugs to the patient to buy outside from the chemist store. Then patient will be like what’s the difference, after wasting my time with the Doctors or Health Workers, they still refer me back to chemist to buy drugs. So there is no need coming again. I rather patronize the chemist. Also other basis amenities like constant electricity, cold chain etc.
7. **What are the reasons for the current utilization rates?**
8. **Are you satisfied with the utilization rates for ANC and delivery? Yes/No. Why?**
9. **What is your view about the state of equipment/facilities/medicines for providing antenatal care and delivery services in your health center?**

**Ans. –** The hospital facilities are so outdated, many of the things used here were procured before the creation of the state in 2006. But thank God for NGOs that have been helping us with so many facilities. I also believe our Governor is doing some soon. And the striking thing is that if you refer any patient to FETHA because there is no equipment, the patient goes home with your referral note to the chemist, TBA or herbal medicine and thus complicates the whole issues. So we rarely refer and just manage with the little ones available.

1. **How do you rate the staff strength of your facility? Are you overworked (understaff)?**

**Ans. –** The staff strength is grossly inadequate. Imagine only one Doctor and four Nurses covering a General Hospital.

1. **Does your health facility offer 24 hour service? YES/NO. Why? Does it affect delivery in the health center? Give reasons.**

**Ans. –** Yes we offer 24hours services only that the available staffs have to adapt a strategy to cover the centre.

1. **What factors do you think make women not to attend ANC in formal health facilities?**
2. **What do you think make the women not to deliver in health facilities even after attending antenatal care there?**

**Ans. –** 7 and 8 has already outlined please.

1. **What are the reasons why women deliver at home?**
2. **What are the reasons women deliver their babies with traditional birth attendants?**

**Ans. –** I have to sound so absorb, the TBAs are creating more problems. I don’t think we need the services of TBAs. In the past, we attempted given them some formal training but who would you educate, someone with no formal education at all, it is not workable. If it is possible to do away with them. Ofcourse, the only way to do away with them is to provide services to all the women in where they live, so they don’t have a need to patronize them. But as it is now, they are still there, they are closer to the women and they cause very severe problems.

1. **What are the reasons why women attend antenatal care in more than one health facility?**

**Ans. –** Yes women attend ANC in more than one place. They jump from place to place, if they go to A and for one reason or the other, the nurse there ask her to buy groves, they jump to another places.

1. **What are the difficulties/constraints in assessing ANC in health facilities?**
2. **What are the difficulties women encounter in delivering their babies in health facilities?**

**Ans. –** 12 and 13

- Poor equipments
- Under staffed, for instance as the only Doctor, you stay from morning till evening and want to go and eat, if any patient comes, he will go back and say there is no Doctor. The few here are overworking themselves.
- Not being well informed.
- Poverty
- Poor road and delays at every stage – Delay at making decision within the family. When the decision is final made to tome to hospital, delay in getting to the hospitals; you then talk about bad roads, poor means of transportation. Also in the hospital, there is delay also in delivery the desire care by the health workers. Pregnancy and childbirth are not emergency situations. So effective ANC of about 4 -5 visits should help the woman in no small ways.

1. **What can be done to overcome these difficulties. (both ANC and DS)**

**Ans. –**

1. Women education and empower – If you educate woman and she have something doing, she will not wait for her husband or any other to give her money before she can access ANC. She may be willing to come to hospital but the decision is made by the man who gives money and where he (the husband) chooses stands.
2. Improve the living conditions of the woman in the villages because these people are really suffering.
3. On the part of the health facility, attitudinal change by health workers. See them as people in need and ensure you help as much as you can. Also always be on duty when you are supposed to be there.
4. On the part of the government, make the hospital be what it is supposed to be. Provide the necessary facilities (drugs and manpower).
5. **What are the roles of health workers in ensuring that women attend ANC and deliver in the health centre?**

**Ans. –**

- - At any place you meet a client, use the opportunity to educate them.
  - Sensitization of the people
  - Ensure you properly educate the woman during ANC especially to ensure healthy baby
  - Go extra mile even in the churches, markets, village meetings etc.

1. **Do husbands have a role to play in determining where women attend ANC and where they deliver their babies? (Yes or No) then explain.**

**Ans. –** Yes they do because the husband that gives the money dictates where the woman does. If he is uneducated he insist the woman uses chemist where she don’t need to pay for card, do lab test etc. On the other way round, educated husbands insists the wife should go the hospitals.

1. **What are the roles of Government at all levels in ensuring that women attend ANC and deliver in health centre?**

**Ans. –** Government should improve their conditions, ensure every woman is educated and proved the facilities needed in the hospitals.

**Conclusion -**

**Interview:**

1. How do rate the attendance of ANC and delivery in the health centre?

(Good, Fair, Poor)

- “It is very poor and there is no delivery at all in the health centre”. People do not always come for ANC and delivery in the centre.

1. What are the reasons for the current utilization rate (very poor) in your health centre?

- People seem to value and prefer TBA and chemist more than health centre. They rather go to these places including travelling to Abakaliki (Urban area by the well to do) for ANC and delivery.
- The woman also said that people complain that the place is too open, i.e there is no privacy and no toilets in the facility.
- Another reason is because of the shortage of staff and absence of the available staff in the night hours when majority of women usually deliver. This is because the same person that worked in the morning and afternoon cannot be available to work at night.

1. Are you satisfied with the utilization rate? Why?

- No, because people coming here for ANC and delivery should be more than what we are seeing.

1. What is your view on the state of equipment’s/ facilities or medications for providing ANC and delivery services in your centre?

- We do not have enough equipments/ facilities, we have only little equipments. And there is no apartment (ward) for women that delivered to stay. That is to say that the place is grossly inadequate.

1. How do you rate the staff strength of your facility, are you overworked?

- There are no staff members. Therefore am overworked, as the only trained officer working with 3 untrained volunteers from the community.

1. Does your facility offer 24hrs service? Yes or No and why?

- No, because there are no enough staff and even if there are, no much people to attend to. So we stay till 6pm, unless in some cases when you have a patient that keeps you until night hours. This affects the 24hrs service in the centre.

1. What factors do you think make more women not to attend ANC in formal health centre?

- I think it’s because they feel they will be charged much. But we are not charging the way they think.

1. What factors make women not to deliver in health centre even after attending ANC there?

- Because they want to go to a place where there are Doctor’s, and those who are afraid they might be charged go to a place where they can deliver free of charge. i.e their homes or TBA’s.

1. What are the reasons women deliver at home?

- From the report we got, they said during night labour before they could come out and go to health centre they would have delivered at home.

1. What are the reasons why women deliver with TBA’s?

- Because of belief and what they were told by their mothers, or relatives, that TBA’s are former place for delivery and that it is easier there than in hospitals. This is because TBAS have been delivering people for ages. They have

1. What are the reasons women attend ANC in 2 places?

- The women told us that they register ANC in different places so that during labour, they can go to the nearest place for delivery.

1. What are the difficulties/ constraints in accessing ANC and delivery services in your facility?

- There is no difficulties on peoples side, the difficulty is on workers side, i.e they are no enough staff to carry out the work. There are no equipments and people complain that the place does not protect their privacy. The facility is not adequate.

1. What do you think can be done to overcome these difficulties?

- They should be MCI to go into the villages and inform the people of the importance of coming to the health centre for delivery. Also inform them of the danger associated with delivering outside the hospital such as contacting infections and other diseases.

**Interviewer**: Who are the MCI?

- MCI are those who go into the villages or homes informing people of the importance of delivering in hospitals and the dangers of delivering without trained healthcare providers.

1. What are the roles of health workers in ensuring that women attend ANC and delivery in health facilities?

- Community mobilization: workers should mobilize the people including outreaches and home visits which we are also doing, telling people about coming to get card for ANC and delivery services. But all these things seems not working because our people need to be forced in certain things. It would be better if there are force-body that will be going around the community to ensure that pregnant women have their ANC cards and attend ANC.

1. Do husbands have a role to play in ensuring that women attend ANC?

- Men (husbands) have a lot of role to play, such as gi6 approval to their wives for ANC and making available the money and other requirements needed for ANC. But it’s obvious that some men do not assist their wives for ANC, some of them will tell their wives to go if they have the money. In other words, they should approve or support their wives financially.

1. What are the roles of the government at all levels in ensuring that women attend ANC and delivery in health centre?

- Government should recruit staff to work in the health centres to enable 24hrs service.
- They should also provide facilities that will make people feel comfortable such as toilets, water supply, electricity etc.
- They should also provide enough equipments.

Conclusion:

**Introduction:**

**Question 1:** How do you rate the utilization of ANC and delivery services in your facility?

**Ans: -** Poor.

**Question 2:** What are the reason for poor utilization?

**Ans:** - They believe in taking herbal drugs for pregnancy related issues. Also they believe that the hospital price (bills) are too much for them to afford due to poor financial status. So they prefer to go to chemist, herbal homes and still some do not know the importance of coming to health facility.

**Question 3:** Are you satisfied with utilization rates?

**Ans: -** No.

**Interviewer:** Why?

We are here in the hospital to save (pregnant women) life. When the people don’t come and bad thing happened to them or their babies, it pains my heart. This community is thickly populated with women of child bearing age but they don’t come to H/C for ANC And delivery.

**Question 4:** What is your view about equipment/ facilities &medication for ANC and delivery services in your centre?

**Ans:** - There are no equipment’s to handle the little ones that are coming. We are suppose to have delivery kit such “amber bag”, in case if a woman delivered and she is having issues, forceps, vacuums and other tools are supposed to be available but we don’t have them. The ones we are using here are the ones we used our money to buy.

**Question 5:** How do you rate the staff strength of your facility? Are you over worked?

**Ans**: - The staff here is not enough. It is only one trained staff, one junior staff and one volunteer. In a place whereby one trained staff is managing the person cannot work in the morning, afternoon and night. It will be an issue if a woman wants to give birth during the night, so you cannot blame people when they go to other places that might have like 3 trained staff that run the hospital day and night.

**Question 6:** Does your facility offer 24 hours service. Yes or No & why?

**Ans:** - We are supposed to run 24hours service but due to the shortage of staff we don’t. However, in a case whereby we have a patient during the day and the woman is on labour, you cannot leave the woman to go so we sleep over. The 24 hours service is affected because there are no enough staff, there are rooms to stay but not equipped. We manage to sit on benches.

- Delivery is affected in the health center because there are no good accommodation for the staff. At least if the staff should be living there, people will be coming at any time of the day.

**Question 7:** What factor do you think makes women not to attend ANC in formal health centre?

**Ans: -** Maybe distance; if their place is far from the health facility. Again is money, if the person does not have the money to come to the hospital, because I could remember one woman about 8 months pregnancy was complaining of body scratching (all over the body) and her baby feeling uncomfortable. I asked her if she has registered with the health facility or any hospital but she said no that her husband said there is no money for hospital. I told the woman to invite the husband, so when the husband came I tried to advise him of the importance of going to the hospital and that the money involved is not much. When they finally went, they found out that the baby is not normal, but doctors did everything possible and it went successful.

- Also fear: they are afraid of going to the hospital especially when you refer them. They complain of nurses and doctor’s behaviour.

**Question 8:** What factors make women not to deliver in health center even after attending ANC there?

**Ans: -** The reason is because of labour time. There are some people you will give appointment for ANC, but they feel since nothing is wrong with them, they are in no pain therefore no need of coming for ANC. So during labour before they will be taken to the health centre they would have delivered at home or nearby places. Or maybe she complained of waist pain and decides to go for native medicine,or TBA but on getting there they will be told that it is labour and in the process she will deliver there.

**Question 9:** What are the reasons women deliver at home?

**Ans:** - Some deliver at home due to the distance of the hospital to their home.

**Question 10:** What are the reasons women deliver with TBAs?

**Ans:** - Advise from other people. They are told that traditional medication is better. Even though some combine both traditional and English medication. Also for those women that have contracted pelvis they believe they will be given medicine that will help them delivered their babies during delivery and there will be no operation. They believe since their neighbours or friends delivered there safely that they will also deliver safely.

**Question 11:** What are the reasons women attend ANC in more than 2 places?

**Ans: -** Because of fear, so that if there is any problem let them not be blamed why the person do not have card with the health facility. Therefore they will have to register with TBA, health centre, or any other health facility

**Question 12/13**: What are the difficulties in accessing ANC and delivery services in your facility?

**Ans: -** People find it difficult to access health facility that is far from their places.

Another constraint is money; anybody that has money will be in a haste to go to hospital, but in a situation where there is no money it will be difficult to even transport themselves to the hospital.

**Question 14:** What do you think can be done to overcome these difficulties?

**Ans: -** To employ more health workers.

-To provide accommodation.

-To provide hospital equipments, even drugs at the minimum price. Although everything cannot be free but at least the price should be at minimum.

-And to ensure that every community have health centre.

**Question 15:** What are the roles of health workers in ensuring that women attend ANC and delivery in health facilities?

**Ans: -** Our roles are that since we are working in rural area, so we are doing home visits, outreach to people. For example we visit a place this week and another week we should visit another place, giving immunization and other health services that will make them easy to access.

**Question 16:** Do husbands have a role to play in determining where women attend ANC and delivery?

**Ans: -** In terms of money, it is the husband that will provide it.It is a thing of joy if the husband’s should assist the women in the hospital, for any provisions and ensure that the women have a good nutrition when she is pregnant. In fact the role of a man is much appreciated if they do it.

**Question 17:** What are the roles of government at all levels in ensuring that women attend ANC and delivery in health centre?

**Ans: -** Government should provide the necessary hospital equipment’s. Employ workers (health worker) that will be working in the health facilities. If hospitals are built and nobody is working there, it does not mean that the hospital is working. And that the hospital bills should be at minimum rate.

**Conclusion:**

Thanks.

Introduction

1. How do you rate the attendance of ANC and delivery in your facility? (Good, fair or poor).

- It is just good.

1. Who do you think is responsible for the current rate?

- So many factors contributed to that.

1. The services we are giving to them.
2. The attitude of the health workers.
3. The hospital is located in a strategic place where so many people can access it.
4. There is no time you will come to the hospital and will not see anybody to attain to you. And we do not run out of stock of the drugs they need.
5. Are you satisfied with the utilization rate?

- Yes, in a primary health care the target for delivery is at least 8 in a month, but as you can see last month we had 35 deliveries. And the average antenatal attendance here is about 150. Like last month we had about 61 new ANC (new people registered for ANC), excluding the people coming for the second, third ANC visit. So if you calculate all of them together you will be having average of 150 in attendance per month.

1. What is your view about the state of equipment’s in your facility?

- We have enough equipment’s at least for labour and delivery. We are well equipped with standard ANC booklet and delivery. In fact, not only government that are helping us but also the NGOs like NCSP are trying for us here. If you go to our labour rooms it is equipped in such a way that nobody enters there without being attracted to deliver there.

1. What is your view about your staff strength?

- We have a little challenge there because it has been long they employed people. However, with the little we have we are managing ourselves very well. The work is actually tedious and we are overworked because in my nursing school I was thought of the ratio of patient per nurse, but here we have more than that per nurse. We have 2 registered nurses and about 6 chores.

1. Does your facility offer 24 hours service?

- Yes, if you have been coming here you will notice that day and night we are here taking care of people.

1. What factors do you think make some women not attend ANC and delivery in health facility?

- Poverty.

Cultural background of people make them not to take ANC so important than even going to TBAs.

1. Are there people that still deliver with TBAs?

- Well, I cannot call them TBAs but another thing is ignorant because some people that are not coming to facility does not know the difference between a trained nurse, a doctor, a place where you can get a nurse midwife trained than all those mushroom private centres. I don’t know whether to call that one TBAs because we have so many quacks around this area. And these people what they do is that if they can’t do virginal examination to ascertain whether the person is in labour, they refer that person to come and see me. Once I noticed that you are in labour, they will say that someone is taking care of them at home and then go back home. And when there is complications they refer them back to the hospital. So one of the factors that make people not attain ANC in the hospital is ignorance.

1. What are the reasons why some deliver at home?

a. Poverty joined with ignorance, because the little money they would have paid in the facility they came to pay more once there are complications during delivery at home.

b. Some may be because of their cultural background permit them to deliver at home that is more natural than delivering in the hospital.

c. Also religious practice affect some people. They go to different ministries and prayer houses where they are told of things that could happen when delivered in the hospital. For example a woman refused to pass through their protocols such as rubbing of oil on the stomach and other things that follows in the prayer house. She said “I insisted coming to the hospital because I believe nothing will happen to me and my baby. She later delivered in the hospital without any challenge. For other people who might agree with their practices and advice will stay there to deliver, until when there are complications they are rushed to the hospital.

10. What are the reasons why women attain ANC in more than one place?

a. One of the major reasons is because every pregnancy is not free from risk. For some of them that are taking emergency preparedness, they will go for more than one places to register so that in emergency case they will have someone to attain to them there. We the nurses also encourage them to register in another place in case of complication. For instance here now we don’t have doctor that will operate on them, for those that have had CSC maybe times two.

b. Another reason is that some of them may decide to register in a place close to them in case of labour, they have to enter the nearest place for their delivery.

11. What are the difficulties/constraint in accessing ANC and delivery in your facility?

- The only difficulties is based on distance, people coming from far distance.

12. What do you think can be done to overcome this difficulty?

- For those of them that do have their own car and cannot transport themselves, we normally reduce their bills because of the transport fare.

13. What is the role of health workers in ensuring that women deliver at health center?

a. One is punctuality and another thing is being responsible to their assigned duties. The health workers here are trying to keep to their shift to make sure that 24hours people come here and see somebody to attain to them”.

b. Another thing is that they create a good relationship with their patients, because when you scud a patient she will not come and will not inform another person of anything good about the person”.

c. Also they should keep to respectful maternity care. The patients are allowed to stay at any position that is comfortable to them during labour unlike before when the nurses shun them anyhow. Now the health workers are trained to respect people’s dignity, all they have, their religious and this creates a very good impression on them.

14. Do husbands have any role to play?

a. Yes the husband should always the supporter to his wife”. They should value their wives and the unborn baby”.

b. In fact in our ANC education we normally show the how a man should always be a supporter to the wife.

c. In the family planning we normally record male involvement, in ANC we also record male involvement. “We normally attain to any women than come with her husband first during ANC”. This special treatment makes the husbands usually come with the wife to the hospital. We make them to feel the importance of supporting their wife from the first day till the day of delivery.

15. What are the role of government in ensuring that women attain ANC and delivery in the hospital?

- Government are doing their best at least public enlightenment that people should be going to ANC and make sure they register in any nearest health facility. Making it as a policy is one of the ways to ensure that women attain ANC and delivery in a health facility.

Conclusion:

**Introduction**:

**Question 1:**

How do you rate the utilization of ANC and delivery services in your Health Centre?

**Answer:** It is good because we have up to 10, 15 or 20 ANC mothers attending ANC in each ANC day and we have it every Wednesdays and Fridays. So we can’t say that it is poor or fair, so it is good.

**Question 2:**

What are the reasons for the current (good) utilization rate as answered above?

**Answer:** It is because we do treat them well they cannot attend ANC in centres where they are harassed or treated as not human beings.

**Question 3:**

Are you satisfied with the utilization of ANC and delivery services in your centre?

**Answer:**  Yes because they do met up with the targeted No for ANC and delivery services in this location, in a month more than 150 ANC visits and average of 20-25 deliveries in our centre.

**Question 4:**

What is the state equipments/facilities/medications for providing ANC and Delivery services in your centre?

**Answer:** Of course yes. We have enough for the capacity of this facility at the level of the healthcare as a PHC.

**Question 5:**

How do you rate your staff strength?, Are you over worked?

**Answer:** As regards to the staff strength, we don’t have enough staff, we need more trained staff and we are believing that the government will post some to us.

**Question 6a:**

Does your facility occur 24-hour service?

**Answer:** Yes because we divided our staff into shifts: morning, afternoon and night to ensure that the place is covered.

**Question 6b:**

Does it affect delivery in the health centre?

**Answer:** It has made the people have confidence on the facility that they are going to met some body to attend to them anytime they come to the centre.

**Question 7**

What factor(s) do you think makes some people not to attend ANC in a formal Health Centre?

**Answer:** We don’t even have many hospitals in this locality, from that Vanco Junction to this place, this is the only health centre that is existing. During raining season, some people living across Iyioukwu River find it difficult to come out due to flooding/rains (ie Access Road). So unavailability of facility close to the people is a factor.

**Question 8:**

What factor do you think make women not to deliver in health centre even after attending ANC there?

**Answer:** Some people still deliver at home but we are trying our best to educate them on the dangers of home delivery. There is poor seeking behavior and age long believe that pregnancy and delivery are not hospital matters

**Question 9:**

Do you know any reason why they deliver at home?

**Answer:** Well! They can give a lot of reason but most times they say that labour started at night when they don’t have any means of coming to the Health Centre. Also many of them have formed it as a habit that after attending ANC, they say they don’t have any problem necessitating hospital, so they just deliver at home with the aid of a TBA or in a TBA Centre.

**Question 10:**

So people still engage the services of TBA even in this place and why?

**Answer:** Of course yes, though we are making efforts to discourage dangers of unskilled delivery. They feel there is no need to come to hospital except you have complications.

**Question 11:**

What are the reasons why women attend ANC in more than one health facility?

**Answer:** I don’t know

**Interviewer: Are you aware that some women attend ANC in more than one health facility?**

**Answer:** Yes because, on booking, if you want to give them Tetenus Toxoid injection, they will say that they have taken it in Mile 4 or Fetha or elsewhere, so the situation is very common.

Also on further inquiry, the women said that they use more than one facility in case of emergency necessitating referral to higher centres. So that they will not be treated as “emergency or unbooked. Also in case of strike action since government ow

**Question 12 &13:**

What are the difficulties or constraints in assessing ANC and delivery services in this centre?

**Answer:** I can’t identify any one except that some people complain of staying more than required (Time wasting) due to lack of lack of adequate staff, in a situation like today where only one person(me) is on duty with many patients to attend to. This is particularly worse with ANC but with delivery, there is no constraints.

**Question 14:**

What do you think can be done to overcome the above constraints?

**Answer:** The government should employ more health workers that are trained to health facilities not only here because many of the health centres if not all are grossly understaffed.

**Question 15:**

What are the roles of health workers in ensuring that women attend ANC and deliver in health facilities?

**Answer:** Public awareness campaigns

- Health education on the need to use health facilities for ANC and delivery.

- Male engagement during ANC so that men will know the benefits of attending ANC and delivery in health facility as to encourage their wives and if need be, ensure that women attend ANC and deliver in health facility.

**Question 16:**

Just as you mentioned above, what other roles do husband have to play in determining where women attend ANC and where they deliver.

**Answer:** Yes because they are the decision makers and in most times providers (suspensor) of the family. So their involvement is needed in determining where women use for ANC and delivery.

Also if a man is part of the pregnancy process, he know what the woman is going through and how to keep.

**Interviewer:** How do you rate male involvement in this area?

**Answer:** For delivery, let me say about 60-70% but in case of ANC, it is still very poor. Not up to 10% of men accompany their wives for ANC. Although we give gifts and incentives to any couple that attend together.

**Question 17:**

What are the roles of Government at all levels in ensuring that women attend ANC and deliver in health facilities?

**Answer:**  The Government has a lot of works to do in this matter. 1^st^ public awareness/enlightment on ANC and delivering matters as well as male involvement.

Also employing more workers and making the facilities more comfortable for workers/patients.

Conclusion:

**Introduction:**

**Question 1:**

How do you rate the utilization of ANC in your centre?

**Answer:** It is very poor – like too poor. We have just a mini hall and in the time past they were not doing delivery in this place. Nothing was happening until I came and partitioned the place, provided one bed and privacy for delivery. The government was promising to come and develop the centre but nothing has been done. So from my own point of view the utilization is very poor.

**Question 2:**

What do you think is the reason for the current poor utilization rate?

**Answer:**  The place is small, not equipped, no toilet and there is no privacy. In fact it does not meet up to urban standard. If it were rural area at least women would have managed because they don’t have many options, but here (urban) people have choices and of course there are so many better places where they have doctors, nurses and well equipped facilities.

From my own point of view, people seem not to patronize primary health centres here in urban because they are usually of below standard.

**Question 3:**

What is the state of the equipment?

**Answer:** The place is not equipped, it is very poor in this 21^st^ century its below standard – not up to expectation. Though it’s a bit better now because of the NGOs like Maternal and Child Survival Programme(MCSP)

that have been helping out, but its still below standard.

**Question 4:**

How the your staff strength?

**Answer:** Its poor, the government is not employing, infact for years now they have not employed workers. Its just me,2 CHEWS and two volunteers that are working here. These volunteers are always available and work to ensure there is 24hrs coverage.

**Question 6:**

Are you over worked?

**Answer:** Yes we overwork, though we are trying our best, we are not giving up. We are managing to make sure there is 24hrs coverage.

**Question 7:**

What factors do you think make women not to attend ANC in a formal health facility?

**Answer:**

- Because of the poor facilities in most government hospitals.

- Secondary, in this locality there are people that still deliver at home, friend’s houses and in churches. So many of them like a women told me when she came for immunization that her Pastor’s wife told her, if she delivers elsewhere other than the church, that something bad would happen to her.

Some also deliver in chemist and anybody that delivered there safely will like to invite another person.

Question 8&9 : What are the reasons why women deliver at home even after attending ANC?

ANS: I don’t know.

**Question 10:**

Do you know about traditional birth attendants (TBAs)?

**Answer:**

TBAs though in town here their practices is quite different from those in the villages. However, people still use them, they call them nurses or doctors, and they go there to deliver their babies. I think why people still deliver with TBAs is because of what they were told and from childhood, they know these people. So they seem to trust them. It is not really because of finance, because in our centre here we don’t charge much, rather they give reasons – like the place is dark, no this and that and that they are not sure whether we will be around, just in a way of giving excuse.

**Question 11:**

Why do some women attend ANC in more than one place?

**Answer:**  We advice people to identify with other hospital that are more equipped and with professional doctors in case of any emergency, because here we don’t have all it takes to take care of them when there are complications. So that when we refer such person they will be attended to. Some women on their own decide to register in both private and government owned hospital in case if government owned hospital like FETHA are on strike.

**Question 12&13:**

What are the difficulties women encounter in assessing ANC and delivering their babies in your centre?

**Answer:** The difficulties include poor facilities, poor access roads.

Another constraint is the waiting time.

**Question 14:**

What do you think should be done to overcome these difficulties?

**Answer:** Government should employ workers, for more than 6yrs now they have not employ workers. At least increase in the number of workers will help overcome the challenge of long waiting. Government should also update health centre, build and equip a standard health centre. And they should be light and supply of other facilities.

**Question 15:**

What are the roles of health workers in ensuring that women attend ANC?

**Answer:** Health workers should go for community immobilization and visits. Let the women know that there delivery in the centre, and that the cost is not much.

**Question 16:**

What role do husbands have to play in ensuring that their wives attend ANC?

**Answer:**

- Husbands are the head of the home, hardly will you see any man that tells his wife to go for ANC having provided money and other supports and she refuse.

- And in our centre, any man that accompanies his wife to ANC, the woman is attended to first before others. So their support reduces the stress on the woman.

- Again, some women find it difficult to take care of themselves but when their husbands are around you see them doing that, taking their drugs and others.

**Question 17:**

What are the roles of government in ensuring that women attend ANC?

**Answer:**  Government should equip health centres. The one here is too local, they should put things in place, water supply, light and other facilities, when they do this people will come.

**Introduction:**

**Question 1:**

How do you rate the utilization of ANC and delivery services in this Centre?

**Answer:** I will say that attendance to ANC is very good but to delivery services and post natal care is only fair. It is just like 30-40% of women who attended ANC end up delivering in this centre.

**Question 2:**

What do you think are the reasons for the above rates of utilization?

**Answer:** I feel sometime, people in this locality feel that when they come to the hospital, they will be charged high hospital bills, so one, it is a mentality problem

2^nd^ – People migrate (travel) that’s why some attend ANC and not end up delivering in this centre.

3rdly – There is poor helath seeking behavior by people in this locality. They prefer to go to TBAs but when complication arises, they come to hospitals.

**Interviewer:** So even in this urban area, people still use TBAs?

**Answer:** Yes – they use TBAs.

4thly – also poverty and ignorance are other factors that limits utilization of ANC and delivery services.

**Interviewer:** What do you think makes more people to attend ANC very well?

**Answer:** I think it is the name of the hospital, people when asked which hospital they registered for ANC, if you call a hospital not well known, people will forge at it. So majority just come to register because of the big name Mile 4 has.

Booking here is perfect more than 1 thousand weekly but afterwards I don’t know what happens.

**Question 3:**

Are you satisfied with the utilization?

**Answer:**  For ANC yes but for delivery services and post natal care no, because they don’t present on time, for instance right now, we have a woman who has had 3 episodes of seizure, the eclampsia is so much, she did not book here and is still unconscious. We delivered baby through vaccum, so the health seeking behavior is still very poor.

**Question 4:**

What is the state equipments/facilities/medications for providing ANC and Delivery services in your centre?

**Answer:** We don’t have problems with drugs, equipments and supplies. They are very efficient (100%).

**Question 5:**

How do you rate your staff strength?, Are you over worked?

**Answer:** As of staff strength we are still coming up, but there is still poor or low manpower although we are managing, so some days the workers overwork but some other days, the workers don’t overwork.

**Question 6:**

Does your facility offer 24-hour service?

**Answer:** Yes, we do.

**Question 7**

What factors do you think makes some people not to attend ANC in a formal Health Centre?

**Answer:** Just like I said before, poverty, some people said they don’t have money to come to hospital, when they come, if you ask them to buy cards, do investigation, ultra-sound, some said they don’t have money even for transportation.

Also if a hospital has a big structure, they only thing they do in by hospital is caesarean section and they fear coming to such facilities. There is high rate of ignorance and lack of knowledge.

**Question 8 & 9:**

What do you think makes some women deliver at home after attending ANC in health facility?

**Answer:** As mentioned above, in adding, fear of big hospital bills makes them to go to TBAs, quack places and complicate issues. For instance, a woman that came last week with retained 2^nd^ twin who ended up loosing the baby, had uterine rupture and hysterectomy because of patronizing chemists, so we still have a long way to do.

We even have a mobile service clinic where we go to these rural areas or difficult to reach areas, book the pregnant women, educate them, give immunizations, drugs and other services and tell them to come to the hospitals if in labour or having any problem.

There is vest awareness about ANC and facility delivery but still we don’t see adequate results, so I think is attitudinal change that we need.

What borders me most is that our PHC and state hospitals are there but no capable trained health personals, the people you will see are JCHEWS and CHEWS and most of them have not been certified as skilled birth attendants. They usually do guess work and at times complicate issues before they refer. Most of them, they don’t even refer on time. They cannot make accurate diagnosis or good treatment. I am even advocating that the government should employ and deplaning stilled personals. So that if a woman cannot afford the services of big hospitals, she can go to PHC or even the general hospitals to serve woman.

**Question 10:**

What are the reasons women deliver with TBS?

**Answer:** As enumerated above.

**Question 11:**

What are the reasons why women attend ANC in more than one place?

**Answer:** I don’t know but I have seen many women do that. They said in case of any problem with any of them, you then refer to the other. They book in government hospital and in private hospital, but it doesn’t make any sense to me. It is usually good to book in a secondary health facility because in PHC private hospitals, they don’t have facilities and equipments to take care of you in case of complicates. That is to say there are no PHC in this locality where they conduct delivery. The only one at Mile 50 is for only minimization, so the state still has a very long way to go.

Another major reason attending ANC in more than one place is in case of strike action usually seen in government facilities.

**Question 12 &13:**

What are the difficulties or constraints in assessing ANC and delivery services in this centre?

**Answer:** No, not in Mile 4, for instance, the woman that has eclimpsia, we didn’t even wait for folder or deposit to be paid before we started care especially when the woman is in labour. There may be constraints of long waiting hours during ANC due to large number of people to be attended to.

Also the centre is located where it is very accessible for people.

**Question 14:**

**Answer: The management of the centre is working to improve on waiting time.**

**Question 15:**

What are the roles of health workers in ensuring that women attend ANC and deliver in health facility?

**Answer:** Increase awareness, keep educating them on the need to present early in hospitals.

Also attitudinal change of the health care providers. Some may be tired and talk anyhow to the patient and she goes back with bad impression of the centre and spread bad information about the centre which in turn affect the overall image of health system.

Here in Mile 4, they taught us how to interact with the patients; also they have done a seminar on health workers for change, so improving our services will help encourage women to use the centres.

**Question 16:**

What are the roles of men/husbands in ensuring that women attend ANC and deliver in health centres.

**Answer:** They have very big role to play, right now we are doing male engagement in ANC, labour and postnatal. Where men are encouraged to follow their wives to ANC, receive health talks and even see doctor together so that if any treatment is given, they will be part of it. And not just give money, they can also remind their wives on things like taking their drugs, next appointment etc. They should not just provide the money, they should be part and parcel ofthe woman’s care.

**Question 17:**

What do you think the government can do at all levels to ensure that women attend ANC and deliver in health facility?

**Answer:**  I have said it before that Ebonyians have poor health seeking behaviours. So the government should deploy trained providers – doctors, nurses and CHEWS into the PHC where the people have close relations.

Also some facilities, the building are dilapidated, no facility and no worker in the centre at night, so women will not want to use such centres. They go to TBAs or chemist shops that are readily available, with relatively better environment.

So government should renovate, put equipments/supplies and employ trained workers and create awareness. They can also do free maternal care services to motivate people.

Conclusion

**Introduction**

**Question 1:** How do you rate utilization of ante-natal care in your facility, attendance of ante-natal care?

**Answer:** It is very good. As patients who attend ante-natal clinic here are numbering 380 – 400 or more daily, I think this number is encouraging.

**Question 2:** What Do you think are the reasons for this wonderful attendance?

**Answer:** I think the reasons are first; the way we attend to patients here is wonderful. And when they come and are treated very well they will go and tell other. Good market sells for itself.

Secondly, there is a program we have in our hospital that came into Ebonyi state that is called **HELLO MAMA.** The program is all about phone messages. In this program (**HELLO MAMA**) their aim is to reduce maternal mortality rate. This program is all about messages and calls and their husbands are being involved. For the fact that the program runs in our clinc, it comes with incentives most times which attracts others. And the type of messages that they send benefits the pregnant women as the messages tell them what they need to know during pregnancy. They also tell them things that they should expect to happen to them which when they start seeing it they will not be startled and take precautional method.

**Question 3:**  Are you satisfied with the rate of utilization?

**Answer:** Of course yes. Many of them make use of our hospital.

**Question 4:** How is your state of equipment in this facility?

**Answer:** Very well equipment. Everything we need, we get if it is available, even some that become faulty are replaced.

**Question 5:**  What of your staff strength?

**Answer:** It is God that is helping us because we are 16 nurses nursing these patients average 380 -400 each day. If not God’s help we will be overwhelmed by the work.

**Question 6:** Do you run 24yrs service?

**Answer:** Yes,

**Question 7:** Do you have any idea why some women don’t attend antenatal care in formal hospital?

**Answer:** Some of them wants quick services, nobody will talk to them. Like here, before the doctor will attend to them, we give them health talk on how to manage their pregnancies, signs and symptoms of labour, things that can male their pregnancy. But in private hospitals, they just go and see the doctor and come out. Some of them deem it that they waste much time in teaching hospital, some measure the money we take, even though they don’t know that the way you manage a patient is according to the issue a patient has.

**Question 8:** Is it the same number of women, that come for antenatal here that came for delivery?

**Answer:** Majority of the people that come for ante-natal deliver here. For the people that do not come is the same ignorance and poverty that cause it. At times, if it is not because of ignorance or poverty it’s because some of them come from far places. Some will be because of lack of fund.

Anybody that comes for ante-natal here cannot deliver at home. They do deliver either at the private hospital or health centre closest to them because of the teaching we give them here.

**Question 9:** In this locality, are there people that use TBAS?

**Answer:** No; rather they use private hospitals and maternity homes. It is the village we here of TBA’S.

In some maternity homes they go to deliver, most of them are not equipped and not trained. When they go there some of them may still come back because of the complicated cases they see in the maternity homes.

**Question 10:** Are you aware that some women attend antenatal care in more than one place?

**Answer:** Yes, because of them coming from far places. They will also register in nearby places; for ease of delivery, for instance, the case of someone coming from Effiom to FETHA. Again in the case of strike they will not like to be stranded. So they will register in other places.

**Question 11& 12:** Are there some difficulties for some people in accessing ante-natal care and delivery services in your facilities?

**Answer:**  We earlier mentioned waiting time. Again lack of money, ignorance some people think that teaching hapital is for some class of people.

**Question 13:** How do you think we can overcome these difficulties?

**Answer:** With what **HELL MAMA** is doing, I think if they continue like this for 2-3yrs people will be rushing here. Again, giving the people attending health talk and more care always will help solve the aforementioned problem.

**Question 14:** What should health workers do to ensure that women attend antenatal care and deliver in health facilities?

**Answer:** Generally, our approach should be unique, we should carry them like princes and kings so that they will value us. When they need us they will call and we will respond to them. Infact we should make them friends. If we do these we will be advertising ourselves.

**Question 15:** Do husbands have roles to play in determine where women attend ante-natal care?

**Answer:** They should be involved so that some tests will be carried out on them. Even, **HELLO MAMA** send the husbands messages and even call them advising them on how to take care of their wives during pregnancy. **HELLO MAMA** also gives gifts to husbands who come with their wives for ante-natal care.

**Question 16:** What do you think is the role of government to ensure that, if possible all women attend antenatal care in qualified hospitals?

**Answer:** The **HELL MAMA** partners with the federal government and **USAID.** If the government can pick up such programs; I think the women will come out for ante-natal. The government can sponsor this program even when **USAID** is stopping it.

Conclusion:

**Introduction**

**Question 1:** How do rate utilization (Attendance) of ANC and delivery services in your facility.

**Answer:** ANC, well is fair, at least during each ANC day we have up to 5-10 women but it is still not enough but delivery is poor.

**Question 2:** What are the reasons for this current rate?

**Answer:** There are so many reasons, on the side of the hospital, the women and the system itself.

One major factor is that the facility does not meet up with the standard of other health facilities in the town especially private hospitals where a person can just build a decent house or rent 3 bedrooms flat as hospitals. But our facility is a mould house with old roof and nothing looking attractive, so many will not want to come.

Also here is town with big hospitals (centres) like FETHA, Mile 4 etc. so many of them want to use where they have big facilities and many workers. To identify with the “big name”, instead of using the Primary Health Centres around them.

**Question 3:** Are you satisfied with the rate of utilization

**Answer:** To be frank, I am not, because here is a highly populated area that should a good turn up to ANC and delivery services but the reverse is the case. If things are done in the right way, people are meant to register in PHC and if need be, they are preferred to higher centres but they go direct to the apex hospital (Fetha).

**Question 4:** What is the state of the facility, equipment ,supplies, drugs?

**Answer:** The equipment/facility is poor. It is just 3 rooms mood house. Though we have few equipments to conduct simple (uncomplicated) delivery but generally it is not up to standard. Though the government has promised to renovate the place but still, nothing is happening presently. The few equipments we have is from donor agencies like MCSP (Maternal and Child Survival Program).

**Question 5:** How do you rate the staff strength of the facility?

**Answer:**  In this centre, it is only me and 2 other trained personals 1 CHEW and 1 JCHEW with 2 untrained personnel that volunteered to help. This is because for so many years, the government has not employed workers.

**Interviewer**: Are you over worked

**Answer:**  Well, we ensure that we share the duties in a way that one is not too much affected; you come when you are supposed to come and handover when to others to continue. We ensure that there is somebody every time in the centre.

**Question 6:** Do you offer 24-hours services

**Answer:** Yes we do! We share the duty in a way that at every point in time, there is somebody to attend to people, even if the person does not haveth capacity to fully and the person calls on the qualified persons who come and respond, 24 hours this place is always open.

**Question 6:**  What factors do you think make women not to attend ANC in a formal health facility?

**Answer:**  Many of them still have confidence in private hospitals and chemists both qualified and unqualified ones. This is because in some privates, the women will not be asked to give or do investigation (Lab, Altra-sound etc). The women want sharp-sharp things, as they come just check their stomach and give them drugs and they are off.

Also many of them believe so much in where their mothers or sisters or neghours delivered in this neighbourhood. They are no long called TBAs in towns because they are known for that for ages.

Money may not really be the factor here because we don’t really charge more than the “chemist or privates” but because that’s where their faith is. Also the women who are Christians have so much trust on their pastors and prayer ministry leaders who prescribe spiritual activities like fasting, midnight prayers, seed sowing etc over formal ANC and delivery services.

**Question 8:** what factors makes some women not deliver in the hospital even after attending ANC.

**Answer:** Yes many of the women who attend ANC do not come for delivery because they just come for ANC to be sure that all is well and then go to these TBAs, Chemist/Private to deliver. Also many just come for free immunization services available in formal centre.

Also some said that labour started in the night and their deliver at home.

**Question 9:** Why do some still delivers at home?

**Answer:**  As answered above, many who delivered at home are people who decided it long ago and some even arrange for a chemist or “quack nurse” for home delivery. Those practices favour arise which are difficult to control

**Question 10:** what are the reason why many of them still deliver their babies with TBAs.

**Answer:**  This is because, they have confidence in the TBAs. Also TBAs don’t waste their time, they know the TBAs and many people (mother in laws) do campaign for TBAs.

We should also do campaigns for our own health system so that people will know that we are better.

**Question 11:** What are the reasons why women attend ANC in more than one facility?

**Answer:**  Yes it very rampart this days, they said its because of fear of emergency. If they register here and there they can be attended to many of them in case. Also they want to compare the type and quality/price of services offered in the different centres and then decided where to deliver. Also they said they register in government and private in case of strike usually in government hospitals.

**Question 12 and 13:** What are the difficulties/constraints in accessing ANC & delivery services in this centre?

**Answer:** No difficulty because the centre is located close to the LGA headquarters. There is access road, so no as of delivering, they are afraid to come in the night in case NEPA takes light because there is no standbye generator.

**Question 14:**  How to overcome these constraints

**Answer:**  Appeal to the government to renovate the place and provide necessary facilities needed for a modern PHC in an urban area such as this place.

**Question 15:** What do you think that Health workers should do to ensure women attend ANC and deliver in Health facility

**Answer:** The health workers should package the system. Even me, if am pregnant again, I will not deliver in this type of place no matter what. Please we should upgrade the facility and services we render. This is urban (town) people are exposed and enlightened and such expect better services/facilities in government hospitals.

- The few patients what come should be treated well with dignity and respect. So that they go back happy and speak good of the facility. We are the image makers of the health system and not just the centre you are working. If we loose them, they run into the hands of quacks.
- Talk good of the hospital (health system) where you live, in you church, women meetings (August Meeting is coming/use I use the opportunity to campaign for facility delivery.
- Home visits and enlightment programs are also necessary.
- Efforts should be focused more on delivery in the presence of skilled attendants.

**Question 16:** What are the roles of husbands (male) in determining where women go for ANC a delivery?

**Answer:**  Yes because they are the head of the family and decision makers of the home. And whatever they ask they wives to do, that they must do. And delivery services by all means.

**Question 17:** Roles of the government

**Answer:**  Government should try and keep their promises, they are doing well in enlightment programmes through radios but they should improve on it.

More importantly, government should set a monitoring team to monitor who is qualified to own and run a private hospital and the services rendered by “chemist shops”, who is qualified to own a chemist and conduct deliver so government should monitor closely the activities of small or big chemist or private hospitals. So that mative doctors will not be handing and complicate issue for us.

**Conclusion**
