# Supplementary material for: Exploring providers’ perceived barriers to utilization of antenatal and delivery services in urban and rural communities of Ebonyi state, Nigeria: A qualitative study
Source: PLoS One. 2021 May 20;16(5):e0252024. doi: 10.1371/journal.pone.0252024 (PMC8136846; doi:10.1371/journal.pone.0252024)
Supplement: Attachment — Submitted filename: Response to Reviewers.docx [file pone.0252024.s003.docx]

PONE-D-20-30363
Exploring providers’ perceived barriers to utilization of antenatal and delivery services in urban and rural communities of Ebonyi state, Nigeria: a qualitative study
PLOS ONE

Dear Dr. EDMUND NDUDI OSSAI,

Thank you for submitting your manuscript to PLOS ONE. After careful consideration, we feel that it has merit but does not fully meet PLOS ONE’s publication criteria as it currently stands. Therefore, we invite you to submit a revised version of the manuscript that addresses the points raised during the review process.

Please submit your revised manuscript by 10 January 2021. If you will need more time than this to complete your revisions, please reply to this message or contact the journal office at plosone@plos.org. When you're ready to submit your revision, log on to https://www.editorialmanager.com/pone/ and select the 'Submissions Needing Revision' folder to locate your manuscript file.

Please include the following items when submitting your revised manuscript:

- A rebuttal letter that responds to each point raised by the academic editor and reviewer(s). You should upload this letter as a separate file labeled 'Response to Reviewers'.
- A marked-up copy of your manuscript that highlights changes made to the original version. You should upload this as a separate file labeled 'Revised Manuscript with Track Changes'.
- An unmarked version of your revised paper without tracked changes. You should upload this as a separate file labeled 'Manuscript'.

If you would like to make changes to your financial disclosure, please include your updated statement in your cover letter. Guidelines for resubmitting your figure files are available below the reviewer comments at the end of this letter.

If applicable, we recommend that you deposit your laboratory protocols in protocols.io to enhance the reproducibility of your results. Protocols.io assigns your protocol its own identifier (DOI) so that it can be cited independently in the future. For instructions see: <http://journals.plos.org/plosone/s/submission-guidelines#loc-laboratory-protocols>

We look forward to receiving your revised manuscript.

Kind regards,

Sharon Mary Brownie
Academic Editor
PLOS ONE


Additional Editor Comments 

The review process has identified very significant issues that require attention within your manuscript if it is to be considered for publication. Of concern, your qualitative quotes do not support your findings. For a returned manuscript please ensure that you include a completed 32 step COREQ checklist of the quality reporting of qualitative research https://www.equator-network.org/reporting-guidelines/coreq/ Please also include a table addressing each point raised by the reviewers.

Journal Requirements:

When submitting your revision, we need you to address these additional requirements.

1. Please ensure that your manuscript meets PLOS ONE's style requirements, including those for file naming. The PLOS ONE style templates can be found at

https://journals.plos.org/plosone/s/file?id=wjVg/PLOSOne_formatting_sample_main_body.pdf and

https://journals.plos.org/plosone/s/file?id=ba62/PLOSOne_formatting_sample_title_authors_affiliations.pdf

**Author response: This has been complied with.**

2. We suggest you thoroughly copyedit your manuscript for language usage, spelling, and grammar. If you do not know anyone who can help you do this, you may wish to consider employing a professional scientific editing service.  

Whilst you may use any professional scientific editing service of your choice, PLOS has partnered with both American Journal Experts (AJE) and Editage to provide discounted services to PLOS authors. Both organizations have experience helping authors meet PLOS guidelines and can provide language editing, translation, manuscript formatting, and figure formatting to ensure your manuscript meets our submission guidelines. To take advantage of our partnership with AJE, visit the AJE website (http://learn.aje.com/plos/) for a 15% discount off AJE services. To take advantage of our partnership with Editage, visit the Editage website (www.editage.com) and enter referral code PLOSEDIT for a 15% discount off Editage services.  If the PLOS editorial team finds any language issues in text that either AJE or Editage has edited, the service provider will re-edit the text for free.

Upon resubmission, please provide the following:

- The name of the colleague or the details of the professional service that edited your manuscript
- A copy of your manuscript showing your changes by either highlighting them or using track changes (uploaded as a *supporting information* file)
- A clean copy of the edited manuscript (uploaded as the new *manuscript* file)

**Author response: Thanks for the observation**

**The manuscript has been reviewed by a senior colleague and research partner, Dr. Chinawa Josephat N.**

**He is an Associate Professor of Paediatrics, Department of Paediatrics, College of Medicine, University of Nigeria, Enugu Campus**

3. When reporting the results of qualitative research, we suggest consulting the COREQ guidelines: http://intqhc.oxfordjournals.org/content/19/6/349. In this case, please consider including more information on the number of interviewers, their training and characteristics. Moreover, please provide the interview guide as a Supplementary File.

**Author response: Thanks for the guidance. The manuscript you referred me to was remarkably good, very helpful and has become a good source of reference.**

4. In your Data Availability statement, you have not specified where the minimal data set underlying the results described in your manuscript can be found. PLOS defines a study's minimal data set as the underlying data used to reach the conclusions drawn in the manuscript and any additional data required to replicate the reported study findings in their entirety. All PLOS journals require that the minimal data set be made fully available. For more information about our data policy, please see http://journals.plos.org/plosone/s/data-availability.

Upon re-submitting your revised manuscript, please upload your study’s minimal underlying data set as either Supporting Information files or to a stable, public repository and include the relevant URLs, DOIs, or accession numbers within your revised cover letter. For a list of acceptable repositories, please see http://journals.plos.org/plosone/s/data-availability#loc-recommended-repositories. Any potentially identifying patient information must be fully anonymized.

Important: If there are ethical or legal restrictions to sharing your data publicly, please explain these restrictions in detail. Please see our guidelines for more information on what we consider unacceptable restrictions to publicly sharing data: http://journals.plos.org/plosone/s/data-availability#loc-unacceptable-data-access-restrictions. Note that it is not acceptable for the authors to be the sole named individuals responsible for ensuring data access.

We will update your Data Availability statement to reflect the information you provide in your cover letter.

**Author response: The authors have requested for assistance in this regard bearing in mind that this was a qualitative study. Depositing such materials is easier with records from a quantitative data collection method. The authors will be pleased to know what we are to submit and how it will be done. We need your assistance, Sir.**

Reviewers' comments:

Reviewer's Responses to Questions

**Comments to the Author**

1. Is the manuscript technically sound, and do the data support the conclusions?

The manuscript must describe a technically sound piece of scientific research with data that supports the conclusions. Experiments must have been conducted rigorously, with appropriate controls, replication, and sample sizes. The conclusions must be drawn appropriately based on the data presented.

Reviewer #1: Partly

Reviewer #2: Yes

2. Has the statistical analysis been performed appropriately and rigorously?

Reviewer #1: N/A

Reviewer #2: N/A

3. Have the authors made all data underlying the findings in their manuscript fully available?

The [PLOS Data policy](http://www.plosone.org/static/policies.action#sharing) requires authors to make all data underlying the findings described in their manuscript fully available without restriction, with rare exception (please refer to the Data Availability Statement in the manuscript PDF file). The data should be provided as part of the manuscript or its supporting information, or deposited to a public repository. For example, in addition to summary statistics, the data points behind means, medians and variance measures should be available. If there are restrictions on publicly sharing data—e.g. participant privacy or use of data from a third party—those must be specified.

Reviewer #1: Yes

Reviewer #2: Yes

4. Is the manuscript presented in an intelligible fashion and written in standard English?

PLOS ONE does not copyedit accepted manuscripts, so the language in submitted articles must be clear, correct, and unambiguous. Any typographical or grammatical errors should be corrected at revision, so please note any specific errors here.

Reviewer #1: Yes

Reviewer #2: Yes

5. Review Comments to the Author

Please use the space provided to explain your answers to the questions above. You may also include additional comments for the author, including concerns about dual publication, research ethics, or publication ethics. (Please upload your review as an attachment if it exceeds 20,000 characters)

Reviewer #1: Introduction
• Much of the literature in the introduction focus on maternal deaths, and access and utilization of ANC as mitigations. Authors worked their way deductively from global perspectives to the Nigerian situation. However, the purpose of the study is to explore providers’ perceived barriers to utilization of antenatal and delivery services in urban and rural Nigerian state. Accordingly, authors should situate the study within theories underlying barriers to utilization of ANC and delivery services.
• What exactly is the problem underlying this study? what does this study seek to achieve? How does this study differ from the vast literatures addressing gaps in maternal and child health within and across LMICs including Nigeria?.

• The conceptualization in its current form is not grounded in new and innovative theoretical perspectives beyond what is known on this subject matter.

**Author response: Thanks for the observation. A paragraph has been included that focused on providers perspectives on barriers to delivery of antenatal and delivery services.**

Methodology

• Authors stated: “In the second stage, a list of all health facilities in the selected local government areas was made and ranked based on the number of women that utilized the health facilities for antenatal and delivery services in the last six months based on hospital records. Twelve health facilities, six each in urban and rural communities were selected based on this criterion”. It is unclear what the criterion is and how it aided the selection.

**Author response: The aim here was to select health facilities which were mostly patronized by the women for antenatal and delivery services. Based on the level of utilization, it is expected that this will impact on the experience of the providers. The authors were of the opinion that the views of the providers in these health facilities that are well utilized will be more useful than those in health facilities not well utilized.**

Authors stated: “The key providers of antenatal and delivery services in each of the facilities were purposively selected for interview”. Who were these key providers and why were they sampled purposively?

**Author response: Twelve providers pf antenatal and delivery services participated in the study. Nine were Officers-in-Charge of the primary health centers. Two were Chief Nursing Officers of a tertiary health institution and a mission hospital. One participant was a Medical Officer in charge of a General hospital.**

**The selection of the providers was based on their official positions and the roles they play in policy formulation and in the delivery of maternal health services in the health facilities.**

• Several content overlaps and repetitions appear though out the manuscript and especially the methodology. This phrase “pretested interview guide”, for example is overly repeated throughout the methodology.

**Author response: Thanks for the observation. Corrections have been made.**

Findings
• The lack of systematic conceptualization of perceived barriers from the onset has affected the quality of the findings – which are a bit disjointed. Example, thematic areas of the findings included: Utilization of antenatal and delivery services in health facilities; Reasons for good utilization of health facilities for antenatal and delivery services; Reasons for poor utilization of health services for antenatal and delivery services; Reasons women deliver at home and by traditional birth attendants; Constraints to use of health facilities for antenatal and delivery services; and How to improve utilization of antenatal and delivery services.

**Author response: These were themes that emerged during the coding process. The authors explored the views of the health providers on issues related to antenatal and delivery services in the study area.**

• I find it extremely difficult collectively relating the thematic areas to barriers of ANC and delivery service use.

**Author response: Barriers to antenatal and delivery services was one of the themes and perhaps the main subject of the interview/ study.**

• Moreover, authors earlier argued that quality of maternal of health services in the study areas is extremely weak. This tend to deter women from utilizing ANC and subsequent uptake of delivery services. To the contrary, the first set of findings suggest that providers are doing their utmost best to provide good quality maternal health services. This reflect in these quotes below:

“We offer quality healthcare to our clients, we ensure that they are satisfied with the services they receive from us. We have also ensured that the attitude of health workers is good” (Female participant, urban)

We practice respectful maternity care. The clients are allowed to stay at any position that is comfortable to them during labour unlike before when health workers shunned such practices. Health workers now respect the dignity of the women and this creates a very good impression on them” (Female participant, urban)

• It can be inferred from the quotes above that authors are evading the purpose of this study. Further, to say in one breath that providers are oriented to good quality care and to turn around and accused the same providers of poor attitude appears to turn this study upside down.

**Author response: Thanks for these comments. Not all the health providers asserted that their health facilities were well utilized for antenatal and delivery services. When it came to constraints, the health workers spoke generally perhaps based on their experiences and other aspects. One remarkable conclusion of this study is the need to train health workers in the study area on quality of care. Indeed, the health workers are doing their best but that best is not good enough.**

**It has already been observed that the health workers have a poor perception of quality of care.**

Ossai EN, Uzochukwu BSC. Providers’ perception of quality of care and constraints to delivery of quality maternal health services in primary health centers of Enugu state. Nigeria. IJTDH. 2015;8(1):13-24.

**The health workers are aware that the women desire quality healthcare. Thus, it may be described as good that in all, the providers were also aware that they (the providers) are not doing enough in taking care of the women. This necessitates the need to train the health workers on quality of care.**

Other comments
While authors have done their best in coming out with this manuscript, the conceptualization, findings and conclusions present no significant new information. There are a plethora of quality theories and evidence produced in response to barriers of maternal and delivery service utilization in sub-Saharan Africa. Such findings produced barriers that cut across poverty, income, demographic and geographic factors, cultural and social norms, religion and many more. You can read more about these studies at: https://scholar.google.com/scholar?start=20&q=why+women+deliver+at+home&hl=en&as_sdt=0,5 and particularly the work of Mrisho and colleagues (2007).

**Author response: Thanks for this information. However, the authors in this study were concerned with the perspective of the health providers on the barriers to antenatal and delivery services in the study area. It may be expected that the health workers will concentrate more on health system factors.**

Reviewer #2: The authors did a good job presenting the barriers around severe maternal mortality in the southeastern part of Nigeria. While the study falls short of a large sample size, it reveals some of the barriers that can be acted upon as helpful and much-needed steps towards a positive intervention for reducing severe maternal mortality in that part of the country.

**Author response: Thanks very much for this observation.**

I have some minor suggested changes, though.

1. The authors could be silent on the majority Christian mentioned in the method section, except if their study shows that religiosity plays a role in the health outcomes.

**Author response: This has been corrected.**

2. In the study design section, correct the punctuation typo before (KII).

**Author response: This has been corrected.**

3. In the study instrument, rephrase the sentence to remove "also" at the end of "use of recorders also.".

**Author response: Corrected.**

4. Combine sections "Study Instrument, Data Collection Methods, Pretesting of instrument" into a single section. There seem to be some repetitions and commonalities.

**Author response: Corrected**.

6. PLOS authors have the option to publish the peer review history of their article ([what does this mean?](https://journals.plos.org/plosone/s/editorial-and-peer-review-process#loc-peer-review-history)). If published, this will include your full peer review and any attached files.


If you choose “no”, your identity will remain anonymous but your review may still be made public.

**Author response:** **Yes. The peer review history can be published.**

**Do you want your identity to be public for this peer review?** For information about this choice, including consent withdrawal, please see our [Privacy Policy](https://www.plos.org/privacy-policy).

Reviewer #1: No

Reviewer #2: No

[NOTE: If reviewer comments were submitted as an attachment file, they will be attached to this email and accessible via the submission site. Please log into your account, locate the manuscript record, and check for the action link "View Attachments". If this link does not appear, there are no attachment files.]

While revising your submission, please upload your figure files to the Preflight Analysis and Conversion Engine (PACE) digital diagnostic tool, <https://pacev2.apexcovantage.com/>. PACE helps ensure that figures meet PLOS requirements. To use PACE, you must first register as a user. Registration is free. Then, login and navigate to the UPLOAD tab, where you will find detailed instructions on how to use the tool. If you encounter any issues or have any questions when using PACE, please email PLOS at [figures@plos.org](mailto:figures@plos.org). Please note that Supporting Information files do not need this step.
