# Supplementary material for: Exploring providers’ perceived barriers to utilization of antenatal and delivery services in urban and rural communities of Ebonyi state, Nigeria: A qualitative study
Source: PLoS One. 2021 May 20;16(5):e0252024. doi: 10.1371/journal.pone.0252024 (PMC8136846; doi:10.1371/journal.pone.0252024)
Supplement: Attachment — Submitted filename: Response to Reviewers.docx [file pone.0252024.s004.docx]

**Response to Reviewers’**

**Editor’s comment**

Journal Requirements:

Please review your reference list to ensure that it is complete and correct. If you have cited papers that have been retracted, please include the rationale for doing so in the manuscript text, or remove these references and replace them with relevant current references. Any changes to the reference list should be mentioned in the rebuttal letter that accompanies your revised manuscript. If you need to cite a retracted article, indicate the article’s retracted status in the References list and also include a citation and full reference for the retraction notice.

**Author response**

**Thank you very much for this observation. The authors were not very sure of the very reference cited in this manuscript that has been retracted. However, the authors reviewed the whole references included in this manuscript and removed reference numbers 3, 5 and 6. They were replaced with new references.**

**We are hopeful that we have resolved this very important issue by this action we have taken. We also plead that we should be guided by the Editor to have this problem resolved assuming our review of the references was not of good effect.**

Additional Editor Comments

The manuscript is much improved in the revised version, however, reviewers have identified the need for a number of further improvements. Please consider each carefully and respond to these in full.

Reviewers' comments:

Reviewer's Responses to Questions

**Comments to the Author**

Reviewer #2: All comments have been addressed

Reviewer #3: (No Response)

Reviewer #4: All comments have been addressed

Reviewer #5: All comments have been addressed

2. Is the manuscript technically sound, and do the data support the conclusions?

The manuscript must describe a technically sound piece of scientific research with data that supports the conclusions. Experiments must have been conducted rigorously, with appropriate controls, replication, and sample sizes. The conclusions must be drawn appropriately based on the data presented.

Reviewer #2: Yes

Reviewer #3: Yes

Reviewer #4: Yes

Reviewer #5: Yes

3. Has the statistical analysis been performed appropriately and rigorously?

Reviewer #2: Yes

Reviewer #3: Yes

Reviewer #4: N/A

Reviewer #5: N/A

4. Have the authors made all data underlying the findings in their manuscript fully available?

The [PLOS Data policy](http://www.plosone.org/static/policies.action#sharing) requires authors to make all data underlying the findings described in their manuscript fully available without restriction, with rare exception (please refer to the Data Availability Statement in the manuscript PDF file). The data should be provided as part of the manuscript or its supporting information, or deposited to a public repository. For example, in addition to summary statistics, the data points behind means, medians and variance measures should be available. If there are restrictions on publicly sharing data—e.g. participant privacy or use of data from a third party—those must be specified.

Reviewer #2: Yes

Reviewer #3: Yes

Reviewer #4: Yes

Reviewer #5: Yes

5. Is the manuscript presented in an intelligible fashion and written in standard English?

PLOS ONE does not copyedit accepted manuscripts, so the language in submitted articles must be clear, correct, and unambiguous. Any typographical or grammatical errors should be corrected at revision, so please note any specific errors here.

Reviewer #2: Yes

Reviewer #3: Yes

Reviewer #4: Yes

Reviewer #5: Yes

6. Review Comments to the Author

Please use the space provided to explain your answers to the questions above. You may also include additional comments for the author, including concerns about dual publication, research ethics, or publication ethics. (Please upload your review as an attachment if it exceeds 20,000 characters)

**Reviewer’s comment**

Reviewer #2: The authors have addressed all comments. Exploring providers’ perceived barriers to utilization of antenatal and delivery services could help reduce severe maternal mortality in that part of the country.

**Author response**

**Thanks for your kind comment**

Reviewer #3: 1. The research question clearly seeks opinion on "barriers" for the utilisation of the Health Facility, whereas the results begin highlighting that the services are well utilised. The paper then goes on to explain how the services rendered can be made more accessible and better but the link to the reader breaks at the first level.

**Reviewer’s comment**

2. What Framework was used for the analysis of the qualitative data?

**Author response**

**This was a qualitative study and the authors have stated that coding was based on themes as they emerged during the coding process.**

**However, in terms of a framework, the concept was to first enquire about the rating of the selected health facilities in terms of utilization of antenatal and delivery services and reasons for such classifications based on the views of the providers. Then the reasons why women deliver at home and with traditional birth attendants. Then the concept of barriers to use of formal health facilities for antenatal and delivery services.**

**We did not stop at this point because bearing in mind that Nigeria bears the greatest burden of maternal deaths globally, the authors sought to find out what could be done to improve the utilization of antenatal and delivery services. This was to serve as the ways of reversing the trend especially by policy makers. We also sought to know** t**he effects of involving husbands in matters related to antenatal and delivery services from the view point of health providers.**

**Reviewer’s comments**
3. Some recommendations to the barriers can be added in the conclusion for a stronger way forward.

**Author response**

**The recommendations have been included.**

**Thanks.**

**Reviewer’s comment**

Reviewer #4: The article is very well written and I enjoyed reading the discussion and comments of reviewers have been addressed

**Author’s response**

**Thanks for your kind consideration**

**Reviewer’s comment**

Reviewer #5: The authors have sought to explore the reasons for barriers for utilization of antenatal and delivery services from the care-providers' perspective in some rural and urban communities of Ebonyi, Nigeria, through this qualitative study. In reply to previous reviewer comment, they have now amply explained the reasons for choosing the particular centers and the providers for interview. However, they have not been able to adequately answer, what new knowledge has this study added to existing literature because the reasons for not attending for antenatal care and delivery at health-care centers in LMICs are already well-known through previous literature. A few more points on how this could be improved would be beneficial add to knowledge.
There is a lot of repetition in the manuscript (discussion) regarding reasons for poor utilisation of health services. This could be stated more concisely.

**Author response**

**Thanks for the observations.**

**The authors explored the views of the providers of maternal health services on the barriers to use of antenatal and delivery services and how to improve the utilization of such services. This is a very important problem in the locality bearing in mind the poor maternal health index in Nigeria. This makes the problems identified as being specific to the locality hence very relevant. It could then be said that the recommendations from this study if applied could be of good effect.**
